# Endoscopic Findings in Patients With Upper Gastrointestinal Bleeding in Ogun State, Nigeria

**DOI:** 10.7759/cureus.23637

**Published:** 2022-03-30

**Authors:** Abiodun C Jemilohun, Kolawole O Akande, Taamaka D Ngubor, Omosivwe Oku, Marion I Ogunmola, Yetunde O Adesuyi

**Affiliations:** 1 Medicine, Benjamin Carson Snr. College of Health and Medical Sciences, Babcock University, Ilishan-Remo, NGA; 2 Medicine, University College Hospital, Ibadan, NGA; 3 Medicine, Babcock University Teaching Hospital, Ilishan-Remo, NGA

**Keywords:** gastrointestinal hemorrhage, peptic ulcer disease, non-variceal hemorrhage, variceal hemorrhage, gastrointestinal endoscopy, upper gastrointestinal bleeding

## Abstract

Introduction

Although the global incidence of upper gastrointestinal bleeding (UGIB) appears to have reduced substantially in the past few decades, acute UGIB still carries significant morbidity and mortality worldwide. There are currently no published data on UGIB in Ogun State, Nigeria. This study examined the endoscopic findings in patients with UGIB in Ogun State.

Methodology

The study was a retrospective cross-sectional survey of patients with UGIB who had upper gastrointestinal endoscopy at three endoscopy centers in Ogun State, Southwest Nigeria, from January 2015 to December 2021. Patients’ data, which included age, gender, and endoscopic findings, were extracted from the endoscopy registers into a spreadsheet and analyzed statistically. Summary statistics included means ± standard deviation for continuous variables and frequencies and percentages for categorical variables. Categorical variables were compared for differences by chi-square test or Fisher’s exact test as appropriate. The statistical significance cutoff was p-value <0.05.

Results

A total of 171 had endoscopy for UGIB during the period under review but 168 had complete data. Out of the 168, 113 (67.3%) were males, giving a male-to-female ratio of 2:1. The mean age of the patients was 52.4 ± 18.1 years, with an age range of 7-85 years. The modal age group was ≥60 years (75; 39.9%). The most common endoscopic finding was peptic ulcer disease (77; 45.8%), followed by esophagogastric varices (27; 16.1%), erosive mucosal disease (25; 14.9 %), portal hypertensive gastropathy (15; 8.9%), suspected malignancies (11; 6.6%), hemorrhagic gastritis (7; 4.2%), gastric antral vascular ectasia (2; 1.2%), and Mallory-Weiss tear (1; 0.6%), respectively. Forty-four patients (26.2%) had no lesion that could explain UGIB.

Conclusion

Peptic ulcer disease was the most common cause of UGIB among our patient population, and the elderly male patients were the most affected.

## Introduction

Upper gastrointestinal bleeding (UGIB) is defined as blood loss within the lumen of the digestive tract proximal to the ligament of Treitz [1]. Although the global incidence of UGIB appears to have reduced substantially in the past few decades due to *Helicobacter pylori (H. pylori)* eradication and improvement in the care of patients with liver cirrhosis, acute UGIB still carries significant morbidity and mortality worldwide [1,2]. Patients with acute UGIB often present clinically with melena, hematemesis, or coffee-ground vomiting and occasionally with hematochezia [1]. 

There is a wide geographical variation in the incidence rates of UGIB ranging from 48 to 160 cases per population per annum, with higher rates among men and the elderly population [1,2]. Since the late 20th century, the management of non-variceal UGIB has evolved from passive diagnostic esophagogastroduodenoscopy (EGD) with medical therapy and interventional surgeries to active therapeutic endoscopy followed by angiographic and surgical interventions if endoscopic therapy fails [3]. Despite the development of new therapeutic measures such as proton pump inhibitors (PPI), therapeutic endoscopy, and surgical interventions, there has been no substantial change in the mortality of UGIB; the mortality still ranges from 5% to 14% [1,2,4].

The etiology of UGIB varies substantially with geographic regions depending on the socioeconomic and demographic characteristics of the population [5]. Causes of UGIB are broadly divided into variceal (esophageal and gastric varices) and nonvariceal (peptic ulcer disease, reflux esophagitis, gastroduodenal erosions, tumors, vascular ectasia, etc.) [1,6]. EGD is the diagnostic modality of choice for UGIB with more sophisticated investigations such as computed tomographic angiography and capsule endoscopy being rarely indicated where endoscopy is inconclusive [4].

Although some single-center studies have been done in different parts of Nigeria [6-10], there is currently no published data on UGIB in Ogun State, Nigeria. Moreover, there is still no robust national data on the subject. Hence, this study aimed at examining the endoscopic findings in patients with UGIB in Ogun State, thereby contributing to the pool of national data. The availability of such information may be useful in healthcare planning, especially for primary and secondary prevention of UGIB. 

## Materials and methods

The study was a retrospective cross-sectional survey conducted at three health facilities in Ogun State, Southwest Nigeria, from January 2015 to December 2021 (seven years): Babcock University Teaching Hospital (BUTH), Ilishan-Remo, Sacred Heart Hospital (SHH), Lantoro, Abeokuta, and Hephzibah Specialist Clinic (HSC), Olorunsogo, Abeokuta. BUTH is a private tertiary health facility, SHH is a private secondary health facility while HSC is a private specialist clinic. SHH was the only healthcare facility where gastrointestinal endoscopy was performed in Ogun State until 2015. Endoscopy services commenced at BUTH and HSC in 2015. These centers were the main endoscopic facilities in Ogun State during the study period.

The study is part of a larger study for which ethical approval was obtained from the Research Ethics Committee of the Ministry of Health, Ogun State, Nigeria (HPRS/381/400). The study was performed in line with the Helsinki Declaration principles. The Board waived written informed consent for the study.

Four endoscopists who had more than five years of endoscopy experience each performed EGD on all the patients with white-light forward-viewing video-gastroscopes in line with international best practices. The procedures were performed with two Karl Storz PKS series video-gastroscopes at BUTH, one Olympus 160 series video gastroscope at SHH, and one Olympus 140 series video gastroscope at HSC. One endoscopist performed procedures at various times at the three centers, two performed procedures at BUTH alone while one performed procedures at SHH alone.

The endoscopy registers of the three centers were searched for patients who had EGD because of UGIB. Patients’ demographic information (age and gender) and endoscopic findings were extracted from the endoscopy reports into a spreadsheet. All male and female patients with UGIB and complete data from the three facilities were included. Each patient was included once. The first endoscopies of patients after UGIB were used while repeat endoscopies were excluded. Patients with incomplete data were also excluded.

Data were analyzed with IBM SPSS Statistics for Windows, Version 22.0 (IBM Corp, Armonk, NY). We summarized age by means ± standard deviation (SD) and categorized it into four groups: ≤19 years, 20-39 years, 40-59 years, and ≥60 years. Categorical variables (age-group, endoscopic lesion, and Forest classification of peptic ulcer disease) were summarized by frequency and percentage. The differences in the gender distribution of the age groups and endoscopic findings were determined by the chi-square test or Fisher’s exact test, as appropriate. The statistical significance cutoff was p-value <0.05.

## Results

A total of 171 patients had EGD for acute UGIB during the period under consideration. Three patients were excluded from data analysis because of incomplete data, leaving 168 patients. Out of the 168, 113 (67.3%) were males, giving a male-to-female ratio of 2:1. The mean age of the patients was 52.4 ± 18.1 years, with an age range of 7-85 years. Patients who were ≥60 years old were the most represented (67; 39.9%) followed by those who were 40-59 years (56; 33.3%), 20-39 years (36; 21.4%), and ≤19 years (9; 5.4%), respectively (Table 1). However, there was no statistically significant difference in the gender distribution of the age groups (p = 0.336).

**Table 1:** Gender distribution of age group among patients with upper gastrointestinal bleeding (n = 168)

Age group in years	Total (n, %)	Male (n, %)	Female (n, %)	p-Value
≤19	9 (5.4)	5 (3.0)	4 (2.4)	
20-39	36 (21.4)	21 (12.5)	15 (8.9)	
40-59	56 (33.3)	42 (25.0)	14 (8.3)	
≥60	67 (39.9)	45 (26.3)	22 (13.1)	
Total	168 (100.0)	113 (67.3)	55 (32.7)	0.336

At endoscopy, 124 (73.8%) patients had lesions that could explain bleeding while 44 (26.2%) had no lesion that could explain bleeding (Table 2). Peptic ulcer disease (77; 45.8%) was the most common finding, followed by esophagogastric varices (27; 16.1%), erosive mucosal disease (25; 14.9%), portal hypertensive gastropathy (15; 8.9%), suspected malignancies (11; 6.6%), hemorrhagic gastritis (7; 4.2%), gastric antral vascular ectasia (2; 1.2%), and Mallory-Weiss tear (1; 0.6%), respectively (Table 2). There were statistically significant differences in the gender distribution of peptic ulcer disease (p = 0.021) and esophagitis (p = 0.023). The other lesions showed no statistically significant difference in gender distribution. The patients without lesions that could explain UGIB either had no mucosal lesion at all or some mucosal erythema without erosion or submucosal hemorrhage. There was also a statistically significant difference (p = 0.002) in the gender distribution of patients with no endoscopic lesions that could explain the bleeding.

**Table 2:** Gender distribution of endoscopic findings in patients with upper gastrointestinal bleeding (n = 168) *Some patients had multiple lesions; ^†^Mucosal erythema with multiple submucosal hemorrhage; ^‡^Some mucosal erythema with no erosion or submucosal hemorrhage. PUD = peptic ulcer disease; PHG = portal hypertensive gastropathy; GAVE = gastric antral vascular ectasia.

Endoscopic finding^*^		Total (n, %)	Male (n, %)	Female (n, %)	p-Value
PUD		77 (45.8)	55 (32.7)	22 (13.1)	0.021
	Gastric ulcer	33 (19.6)	18 (10.7)	15 (8.9)	
	Duodenal ulcer	34 (20.2)	30 (17.9)	4 (2.4)	
	Both	10 (6.0)	7 (4.2)	3 (1.8)	
Varices		27 (16.1)	23 (13.7)	4 (2.4)	0.107
	Esophageal	20 (11.9)	18 (10.7)	2 (1.2)	
	Gastric	2 (1.2)	1 (0.6)	1 (0.6)	
	Both	5 (3.0)	4 (2.4)	1 (0.6)	
Erosive mucosal disease		25 (14.9)	19 (11.3)	6 (3.6)	
	Gastroduodenal erosion	15 (8.9)	9 (5.3)	6 (3.6)	0.801
	Esophagitis	10 (6.0)	10 (6.0)	0 (0.0)	0.023
PHG		15 (8.9)	12 (7.1)	3 (1.8)	0.810
Malignancies		11 (6.6)	9 (5.4)	2 (1.2)	0.557
	Esophageal	1 (0.6)	1 (0.6)	0 (0.0)	
	Gastric	7 (4.2)	5 (3.0)	2 (1.2)	
	Duodenal	3 (1.8)	3 (1.8)	0 (0.0)	
Hemorrhagic gastritis^†^		7 (4.2)	6 (3.6)	1 (0.6)	0.288
GAVE		2 (1.2)	1 (0.6)	1 (0.6)	0.610
Mallory-Weiss tear		1 (0.6)	1 (0.6)	0 (0.0)	0.484
No lesion to explain bleeding		44 (26.2)	21 (12.5)	23 (13.7)	0.002
	Mucosal erythema^‡^	36 (21.4)	19 (11.3)	17 (10.1)	
	No lesion	8 (4.8)	2 (1.2)	6 (3.6)	

Concerning the risk of rebleeding among the 77 patients with peptic ulcer disease, the majority had Forest class 3 (66; 85.7%) followed by Forest class 2C (4; 5.2%), Forest class 2B (3; 3.9%), Forest class 2A (2; 2.6%), and Forest class 1B (2; 2.6%), respectively (Table 3). No patient had Forest class 1A.

**Table 3:** Forest classification of peptic ulcer disease (n = 77)

Class	Frequency	Percentage
1A	0	0.0
1B	2	2.6
2A	2	2.6
2B	3	3.9
2C	4	5.2
3	66	85.7
Total	77	100.0

Regarding therapeutic intervention, five patients with esophageal varices had variceal band ligation while two patients with peptic ulcer bleeding had adrenaline and absolute alcohol injection, making a total of seven patients.

## Discussion

Acute UGIB is a common medical emergency worldwide with significant morbidity and mortality [1]. The mean age of our subjects was 52.4 years. This is comparable to the mean ages of 51.5 years and 47.6 years obtained by Uiagbe et al. in Benin, Edo State [6] and Akere et al. in Ibadan, Oyo State [7], respectively, in Southern Nigeria, but higher than the mean ages obtained from studies in Kano, Kano State [8]; Maiduguri and Gombe, Borno and Gombe States [9]; and Zaria, Kaduna State [10] in Northern Nigeria that were generally less than 44 years. The reason for this disparity is not immediately clear but it may be related to the age distribution profile of the populations.

A breakdown of the ages of the subjects shows that the elderly (≥60 years) patients were the most affected by the disease. This corresponds to findings from other parts of the world as well [2]. Apart from the fact that the common causes of UGIB such as peptic ulcer disease and gastroesophageal varices are also present in the elderly, aging-related cardiovascular and rheumatological conditions that require the use of aspirin, non-aspirin antiplatelets, nonsteroidal anti-inflammatory drugs (NSAID), and anticoagulants make the elderly more prone to UGIB [4].

In the present study, more males were affected than females with a male-to-female ratio of 2:1. Several studies in Nigeria [6,9-11] and other parts of the world [2,12,13] have also shown a higher preponderance of acute UGIB among the male population than the female. The reason for the observed high male-to-female ratio in our study is not clear. However, it could be because the males were able to afford endoscopy more than the females since health services are largely funded out of pocket in Nigeria.

Ogun State is located in the Southwest region of Nigeria. The socioeconomic condition in the state is similar to those of other states in the sub-region and Southern Nigeria in general. In our study, non-variceal hemorrhage was generally more than variceal hemorrhage with peptic ulcer disease topping the list (45.8%). This finding is consistent with findings from previous studies in Southern Nigeria that showed either peptic ulcer disease [6,14] or erosive mucosal disease [7,11] as the most common cause of acute UGIB in the region. However, studies from Northern Nigeria have consistently shown esophageal varices as the most common cause of UGIB in the region [9,10,15].

Whereas our findings at endoscopy are consistent with those of the Western world [2] and some Asian countries like Iran [13] and Saudi Arabia [16] that also found peptic ulcer disease and erosive mucosal disease as the most common causes of UGIB, they contrast with findings from other African countries such as Tanzania [17,18], Malawi [19], Uganda [20], and Zambia [21], and Asian nations like Pakistan [22] and India [12,23] that showed esophageal varices as the most common cause of UGIB. It is generally believed that peptic ulcer disease and mucosal erosion are the most common causes of UGIB in the developed world while variceal hemorrhage is the most common cause in the developing world [2,24]. The high burden of chronic hepatitis, especially from hepatitis B (HBV) infection, and schistosomiasis which cause chronic liver disease (CLD) and portal hypertension are said to be the cause of the disparity in the etiology of UGIB between industrialized and developing countries [10,24].

The reason for the Nigerian North-South dichotomy in the etiology of UGIB is not immediately clear; it could be that the burden of CLD is more in the north than in the south of Nigeria. A recent systematic review and meta-analysis showed that, where adequate data were available, hepatitis B seroprevalence is generally higher in the northern states of Nigeria than in the southern states [25]. Differential access to treatment of chronic infectious hepatitis and schistosomiasis could also play a role.

Also, the level of alcohol consumption and the brands consumed could be partly responsible for the Nigerian north-south disparity in the etiology of UGIB. For example, a cheap locally brewed sorghum-based beer known as Burukutu is consumed widely in Northern Nigeria, especially by people of low socioeconomic status [26]. In addition to the alcohol content, Burukutu contains two other hepatotoxins (aflatoxin and iron) [27,28] and has been shown to have more impact on liver biomarkers than the regular commercial beer [26]. In a study conducted by Okeke et al. in Jos Plateau [28], North-Central Nigeria, 76% of patients with liver cirrhosis drank alcohol significantly, 51% of whom drank Burukutu alone. However, a study conducted in Southwest Nigeria reported that only 35.2% of patients with CLD drank significant quantities of alcohol, none of whom drank Burukutu [29]. These findings suggest that the availability of local alcohol-brewing capacity and cheap brands of alcohol could increase the level of alcohol consumption in a region irrespective of social status. Thus, regional differences could exist in the prevalence of liver cirrhosis and variceal hemorrhage in Nigeria.

Since the prevalence of *H. pylori* is high in Nigeria, irrespective of the region, another plausible factor for the north-south dichotomy in the etiology of UGIB in the country is a disparity in the use of NSAID between the two regions. There could be a preponderance of NSAID use in the south since the south is generally more educated and developed than the north of Nigeria. 

Although EGD is said to have a high yield in identifying the cause of UGIB if performed within 24 hours of the onset of bleeding [9], we could not find the cause of bleeding in 26.2% of our cases. This is rather high compared to 4.8%-11.9% found elsewhere in Nigeria [6,9-11]. The reason for this observation could be the fact that the majority of our patients did not have EGD within 24 hours of the onset of bleeding, either because of a lack of financial wherewithal or they were referred to our centers after they had stopped bleeding. As such, the majority had already received PPI for a reasonable time before endoscopy. Gastrointestinal mucosal lesions could heal quickly because of the rapidity of the gut’s epithelial cell multiplication. Hence, the duration between the onset of bleeding and the performance of EGD influences the likelihood of finding the cause of bleeding [17]. Moreover, we did not consider mucosal erythema without clear evidence of erosion or hemorrhage as a possible source of bleeding as some of the studies cited may have done. Again, the observation that the majority of the patients with peptic ulcers had intermediate (11.7%) to low risk (85.7%) of rebleeding could be adduced to late endoscopy performance and use of PPI.

The retrospective nature of the study and the fact that a reasonable number of the patients were referred to us for endoscopy from other centers after they had stopped bleeding precluded a detailed study of the clinical features and risk factors of UGIB in the patients. Also, the data presented here need to be interpreted with caution because of the relatively small number of procedures performed during the seven-year period under review. Nevertheless, this study is important because it provides the first set of data on upper gastrointestinal hemorrhage from Ogun State, Nigeria.

## Conclusions

Our study showed that peptic ulcer disease was the most common endoscopic finding among patients with UGIB in Ogun State, Nigeria, during the period considered. It also showed that the elderly male patients were the most affected. Given that EGD has a high diagnostic yield when performed within 24 hours of bleeding onset, early referral of patients with UGIB to facilities with endoscopy service is highly desirable as this would enhance prompt identification of the cause of bleeding and application of therapeutic measures where appropriate. This study, again, brings to the fore the north-south dichotomy in the etiology of UGIB in Nigeria. Therefore, a comprehensive nationwide study is desirable to determine the burden of the disease, and the reasons for the north-south dichotomy observed in its etiology in the country. 
